# Development of an Experimental Model for Analyzing Drug Resistance in Colorectal Cancer

**DOI:** 10.3390/cancers10060164

**Published:** 2018-05-28

**Authors:** Mohamed Elbadawy, Tatsuya Usui, Hideyuki Yamawaki, Kazuaki Sasaki

**Affiliations:** 1 Laboratory of Veterinary Pharmacology, Department of Veterinary Medicine, Faculty of Agriculture, Tokyo University of Agriculture and Technology, 3-5-8 Saiwai-cho, Fuchu, Tokyo 183-8509, Japan; mohamed.elbadawy@fvtm.bu.edu.eg (M.E.); skazuaki@cc.tuat.ac.jp (K.S.); 2 Department of Pharmacology, Faculty of Veterinary Medicine, Benha University, Moshtohor, Toukh, Elqaliobiya 13736, Egypt; 3 Laboratory of Veterinary Pharmacology, School of Veterinary Medicine, Kitasato University, Towada, Aomori 034-8628, Japan; yamawaki@vmas.kitasato-u.ac.jp

**Keywords:** 3D cell culture, colorectal cancer, drug resistance, cancer stem cells

## Abstract

Colorectal cancer (CRC) is one of the most common cancers, for which combination treatment of chemotherapy is employed. However, most patients develop drug resistance during the course of treatment. To clarify the mechanisms of drug resistance, various research models have been developed. Recently, we established a human CRC patients-derived three-dimensional (3D) culture system using an air-liquid interface organoid method. It contained numerous cancer stem cells and showed resistance to 5-fluorouracil and Irinotecan. In this review, we introduce conventional and our established models for studying drug resistance in CRC.

## 1. Introduction

Colorectal cancer (CRC), also known as colon cancer, rectal cancer, or bowel cancer, is one of the leading causes of cancer-related fatalities worldwide [1,2,3,4,5,6,7,8]. CRC metastasis accounts for 40% to 50% of newly diagnosed patients, which is associated with high morbidity [9]. Despite therapeutic advances in the treatment of CRC, the prognosis for patients with metastatic CRC remains poor. A few cancer stem cells (CSCs) are contained in the CRC tissues, which is assumed to possess the capacity to metastasize and to cause resistance to anti-cancer drug treatment [10]. Nevertheless, the detailed mechanisms underlying the relationships between CSCs and resistance to chemotherapy are not fully understood due to the lack of a proper experimental model to examine them.

## 2. Treatment of Colorectal Cancer (CRC)

Currently, surgery and chemotherapy are the two main treatment options for CRC, depending on the cancer stage and location, as well as an individual condition of the patients [11]. Around 25% of CRC patients are incurable at diagnosis, and 50% of patients who undergo surgery will develop metastasis. Chemotherapy is often used as an adjuvant either before surgery in treating CRC to slow tumor growth, shrink tumor size, and reduce the possibility of metastasis or after surgery for patients with advanced CRC [12]. The primary therapy for advanced CRC depends on the administration of fluoropyrimidines (5-fluorouracil (5-FU) or capecitabine) in combination with oxaliplatin or topoisomerase I inhibitor (Irinotecan). Recently, monoclonal antibodies targeting the epidermal growth factor receptor (EGFR), such as cetuximab, bevacizumab, and panitumumab have been proven to be effective in combination with chemotherapy or as single agents for the treatment of metastatic CRC [3,13,14]. Although most patients with advanced CRC are initially responsive to combined chemotherapy treatment, the effects are gradually lost due to the emergence of drug-resistant tumor cells, leading to cancer relapse and metastasis.

## 3. Resistance of CRC to Chemotherapy

Some cancers are regarded to be resistant to therapy at the time of drug exposure (innate drug resistance), but others become resistant after an initial response (acquired drug resistance). To date, multidrug resistance in CRC is still an obstacle to successful chemotherapy. Despite advances in chemotherapy, the five-year survival rate remains low [6], and the main reason for treatment failure is supposed to be the acquired resistance to therapy, which occurs in most patients with metastatic CRC [15].

Even molecular targeting therapy against EGFR causes resistance within 3–12 months [13,16], necessitating a change in treatment.

## 4. General Mechanisms of Drug Resistance in CRC

**Figure 1:**
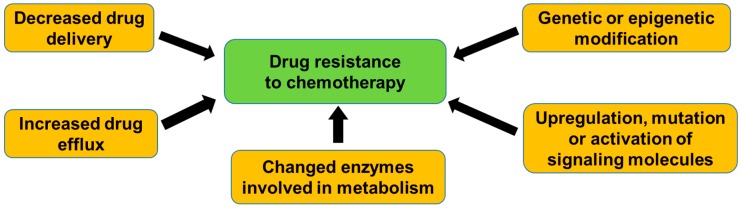
General mechanisms of drug resistance in colorectal cancer (CRC). Drug resistance is caused by multiple mechanisms, such as the decrease in delivery of drug to cancer cells, increase in an efflux out of the cells mediated by ATP-dependent transporters, decrease in uptake into the cells, change in enzymes involved in metabolism, genetic, or epigenetic modifications in the cells, and upregulation, mutation, or activation of downstream of signaling molecules.

Drug resistance in CRC involves multiple mechanisms, such as the decrease in the delivery of drug to the cancer cells, increase in an efflux out of the cells that are mediated by ATP-dependent transporters, decrease in uptake into the cells, or a change in enzymes that are involved in metabolism [17]. On the other hand, resistance can be conferred by genetic or epigenetic modifications in the cells, which alters drug sensitivity [18]. Resistance to targeted therapies also occurs by different mechanisms, including upregulation, mutation, or the activation of downstream signaling molecules by a cross-talk between signaling pathways [15,19] (Figure 1).

In CRC, it was shown that the higher expression level of thymidylate synthase and topoisomerase I are associated with tumor insensitivity to 5-FU and camptothecin derivatives-based therapy, respectively [20]. Glucuronidation, which is involved in xenobiotic detoxification, regulates innate resistance to topoisomerase I inhibitors in CRC cell lines [21]. Resistance to oxaliplatin involves the decrease in a drug accumulation, increase in detoxification, enhancement of tolerance to damage, and alteration in the pathways that are involved in cell cycle kinetics [22].

Since the mechanisms are not fully understood, useful biomarkers to overcome the resistance are hardly found. The only clinically used biomarker is KRAS. Patients harboring a KRAS mutation are excluded from being treated with EGFR antibodies, as they are less likely to benefit from EGFR-targeted treatment [23].

## 5. Regulation of Drug Resistance by Cancer Stem Cells (CSCs)

**Figure 2:**
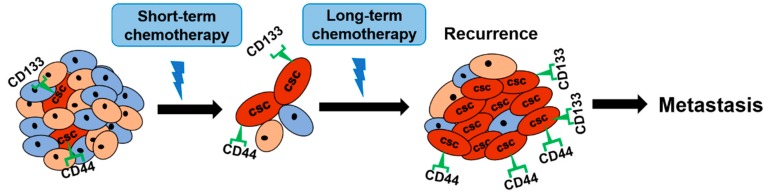
Regulation of drug resistance by cancer stem cells (CSCs). Short-term chemotherapy is effective to most cancer cells but not CSCs. During long-term chemotherapy, CSCs expressing cell surface markers (CD44, CD133) are gradually increased, which might cause tumor recurrence and metastasis.

Cancer cells are heterogeneous in morphology, inheritance, and functions. Among cancer cells, cancer stem cells (CSCs) possess the ability of self-renew, survival, and generation of the tumor. It has also been considered that CSCs might regulate the mechanisms of intrinsic or acquired drug resistance, which leads to the recurrence of progressively invasive and malignant cancers [24,25,26] (Figure 2).

CSCs could be identified by specific cell surface markers, such as CD44, CD24, and CD133 [27]. Several studies showed that these CSC marker-positive cells exhibit more chemoresistant behavior than negative ones. The overexpression of CD44 in tumor cells was strongly associated with therapeutic drug resistance [28]. In the same report, CD133 was overexpressed in human CRC cell lines that were resistant to 5-FU and oxaliplatin [28]. Besides, the number of both CD44 and CD133-positive cells was increased in CRC cell lines after treatment with 5-FU or oxaliplatin [28]. In the same report, both CD44 and CD133-positive cells in HT29 cells showed the increased expression of insulin-like growth factor receptor, which regulated the resistance to anti-cancer drug treatment [29].

Mechanistically, Todaro et al. found that treatment with an IL-4 receptor antagonist or an anti-IL-4 neutralizing antibody strongly enhanced the anti-cancer efficacy of standard chemotherapeutic drugs through selective sensitization of CD133-positive cells. In CRC sphere cultured cells, treatment with anti-IL-4 neutralizing antibody decreased the expression of anti-apoptotic proteins, cellular FLICE-inhibitory protein, Bcl-xL, and PED [30,31]. These studies suggest the possibility that IL-4 become a promising therapeutic target for chemoresistance in CRC.

Data have also shown that Wnt pathway activity could be responsible for the chemoresistance of CD133-positive cells in CRC. Deng et al. demonstrated that 5-FU upregulated Wnt activity in CD133-positive cells [32]. Besides, Dickkopf-1, which is an inhibitor of the Wnt pathway, reduced the proliferation, migration, and invasion of CRC cells through the decrease in the expression of CD133 and LGR5 [33], suggesting that inhibition of Wnt pathway might be a possible solution to the problem of chemoresistance.

These papers imply that CSC-targeting therapeutic strategy might improve drug resistance in CRC patients. Nevertheless, most of the studies were conducted by using cancer cell lines in vitro. More clinical studies using CRC patient-derived CSCs should be performed to clarify the role of CSC markers in chemoresistance.

## 6. Three-Dimensional (3D) Cell Culture Model to Study Drug Resistance in CRC

Gaining insight into the mechanisms of acquired resistance to anti-cancer drugs is critical for the development of novel, rational, and more effective treatment [3]. Since various experimental models can be easily generated by a selection while using anti-cancer drugs in vitro, cellular mechanisms of drug resistance in cancer have been intensively studied [18].

For example, Liu et al. established oxaliplatin-resistant human CRC cell lines by continuous exposure of oxaliplatin [34]. In the established cell lines, cross-resistance to 5-FU, etoposide, cisplatin, vincristine, and epirubicin, but not to paclitaxel, was observed. In the oxaliplatin-resistant cells, the expression of a transporter protein, MRP2, was upregulated, while the expression of P-gp and MRP1 did not significantly change. In the same cells, CD133 was overexpressed while CD44 level remained unchanged. These results suggest that the long-term treatment of CRC cell lines with anti-cancer drugs shows a typical and stably resistant phenotype and it may be used as research models. However, it is difficult for these two-dimensional (2D) cells to reflect cellular heterogeneity and behavior of tissues in vivo. The present review, therefore, will focus on different culture models to analyze drug resistance in CRC.

The past decades have seen the accelerating implementation of three-dimensional (3D) cell cultures in drug discovery [35,36,37]. Recently, 3D primary organoid culture system was established using Matrigel, which could mimic the crypt-like structures of small intestinal tissues [38]. Besides, Matrigel organoid from CRC patients was produced where tumor organoids closely recapitulated properties of the original tumor [39]. These reports suggest a possibility that the technology of Matrigel organoid culture is applicable to personalized therapy for CRC in the near future.

CSCs are affected by a microenvironment, consisting of epithelial and mesenchymal cells, and extracellular substrates [40,41]. To establish a culture system that mimics tumor microenvironment in the 3D culture, Ootani et al. used another type of 3D culture system, called an air-liquid interface (ALI) method [42]. The characteristics of ALI culture is the utilization of double layered collagen gel. Intestinal stem cell-stimulating media is added in the outer well to intrude into the lower layer in the insert well. Tissue fragments are contained in the upper layer and are exposed to air, which may mimic the environment of intestinal tissues and grow organoid rapidly [43]. Since ALI organoid culture consists of epithelial and stromal cells, like co-culture condition [44], it can more reflect the microenvironment in vivo.

**Figure 3:**
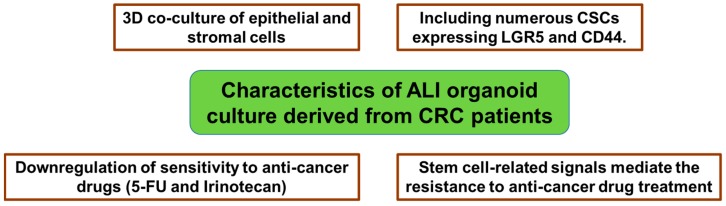
Characteristics of air-liquid interface (ALI) organoid culture derived from CRC patients.

In the previous study, we for the first time established ALI organoids from malignant human colorectal tissues [45]. Tumor ALI organoids consisted of epithelial and stromal cell components and they contained many CD44 and LGR5-positive cells. Interestingly, tumor ALI organoids were more resistant to the toxicity of 5-FU and Irinotecan when compared to CRC cell lines, SW480, SW620, and HCT116. These findings indicate that ALI organoid culture from CRC patients may be useful for examining resistance to chemotherapy in the tumor microenvironment (Figure 3).

Also, we investigated which stem cell-related signaling mediates the resistance in tumor ALI organoids. Combination treatment of Hedgehog signal inhibitors (AY9944 or GANT61) with 5-FU, Irinotecan, or Oxaliplatin upregulated the sensitivity of tumor ALI organoids [46]. These results indicate that stem cell-related signals mediate anti-cancer drug resistance in our established model, and that the model is useful for investigating the mechanisms of drug resistance of CRC (Figure 3).

## 7. Conclusions

The development of experimental model is a key to increase the quality of preclinical research, and to succeed in the development of new therapeutic drugs. In this area, rapid progress has been made to get as close as possible to in vivo situations of human CRC cancers. Even though cell lines and animal models are still indispensable, the 3D culture of CRC cells holds promises for clarifying the more detailed mechanisms of drug resistance and the development of more efficient and safer anti-cancer drugs.
